# The role of ECoG magnitude and phase in decoding position, velocity, and acceleration during continuous motor behavior

**DOI:** 10.3389/fnins.2013.00200

**Published:** 2013-11-01

**Authors:** Jiri Hammer, Jörg Fischer, Johanna Ruescher, Andreas Schulze-Bonhage, Ad Aertsen, Tonio Ball

**Affiliations:** ^1^Bernstein Center Freiburg, University of FreiburgFreiburg, Germany; ^2^CorTec GmbHFreiburg, Germany; ^3^Neurobiology and Biophysics, Faculty of Biology, University of FreiburgFreiburg, Germany; ^4^Epilepsy Center, University Medical Center FreiburgFreiburg, Germany

**Keywords:** brain-machine interfaces, low-frequency component, phase, decoding, Fourier descriptors, multiple linear regression, continuous movement

## Abstract

In neuronal population signals, including the electroencephalogram (EEG) and electrocorticogram (ECoG), the low-frequency component (LFC) is particularly informative about motor behavior and can be used for decoding movement parameters for brain-machine interface (BMI) applications. An idea previously expressed, but as of yet not quantitatively tested, is that it is the LFC phase that is the main source of decodable information. To test this issue, we analyzed human ECoG recorded during a game-like, one-dimensional, continuous motor task with a novel decoding method suitable for unfolding magnitude and phase explicitly into a complex-valued, time-frequency signal representation, enabling quantification of the decodable information within the temporal, spatial and frequency domains and allowing disambiguation of the phase contribution from that of the spectral magnitude. The decoding accuracy based only on phase information was substantially (at least 2 fold) and significantly higher than that based only on magnitudes for position, velocity and acceleration. The frequency profile of movement-related information in the ECoG data matched well with the frequency profile expected when assuming a close time-domain correlate of movement velocity in the ECoG, e.g., a (noisy) “copy” of hand velocity. No such match was observed with the frequency profiles expected when assuming a copy of either hand position or acceleration. There was also no indication of additional magnitude-based mechanisms encoding movement information in the LFC range. Thus, our study contributes to elucidating the nature of the informative LFC of motor cortical population activity and may hence contribute to improve decoding strategies and BMI performance.

## Introduction

Brain machine interfaces (BMIs) are devices that have the potential to restore movement ability in severely paralyzed patients by using neuronal signals to control external effectors. A prominent BMI approach is to directly translate neuronal movement-related activity corresponding to (intended, attempted, or executed) movements into those of an external actuator. This *direct motor decoding approach* has been successfully used for closed-loop motor control with multiple single-unit activity (SUA) in monkeys (Carmena et al., 2003) and humans (Hochberg et al., 2006; Collinger et al., 2013) and also with electrocorticography (ECoG) (Yanagisawa et al., 2011; Milekovic et al., 2012), utilizing a *low frequency component* (LFC) of measured neuronal population signals (Milekovic et al., 2012). A growing number of offline studies [reviewed by Waldert et al. (2009)] indicate that the LFC contains substantial information about a wide range of movement parameters.

**Figure 1:**
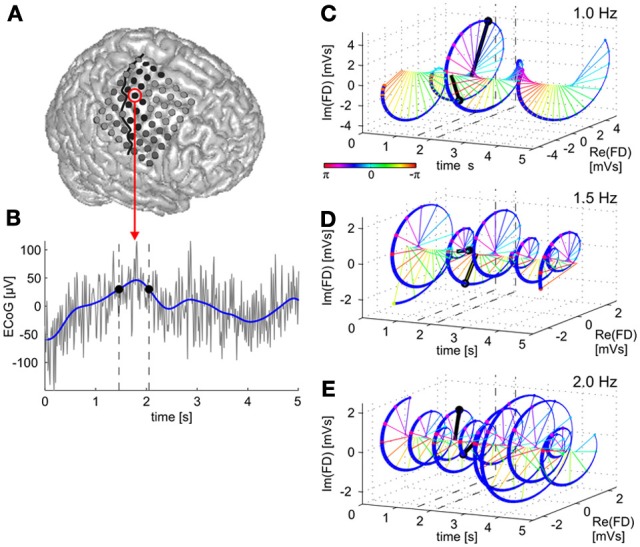
**Time domain vs. time-frequency domain representations of the ECoG low-frequency component (LFC).** A raw brain signal recorded from a site over premotor cortex **(A)** is shown in **(B)** (gray curve). The LFC in the time domain is derived by low-pass filtering (blue curve). Two LFC values with the same amplitudes are indicated by black dots at their respective time points indicated by dashed vertical lines. The LFC data can be transformed to an equivalent, complex valued, time-frequency representation, given by the time course of magnitude and phase values of oscillatory components at different frequencies within the LFC band. In **(C)**, the time course of the 1.0-Hz oscillatory component of the brain signal is shown. Data vectors at the same two time points as indicated in **(B)** are drawn in black, signal magnitude corresponds to the length of a vector and phase to its angle (color-coded) in the complex space. Note that both magnitude and phase at the two selected time points in **(B)** are now different. The 3D figure shows time (*x*-axis) and real (*y*-axis) as well as imaginary (*z*-axis) components of the signal's Fourier descriptors (FDs). **(D,E)**—the same for the 1.5-Hz and 2.0-Hz oscillatory components, respectively. In the present study, we examined trajectory encoding and decoding of ECoG signals based on such time-frequency representations to determine the contributions of magnitude and phase information, respectively.

Previous studies have suggested, but not quantitatively tested, that phase information contained in the LFC signal might play a more crucial role in LFC-based motor decoding than magnitudes (Jerbi et al., 2007; Waldert et al., 2008; Ball et al., 2009, see Figure 1). A related, yet unresolved question is which frequencies—out of the range used in previous LFC studies—are most informative. An examination of these issues would require a quantitative comparison of the accuracies of phase- and magnitude-based decoding—this is currently lacking. There is mounting evidence that the phase of neuronal activity in the lower frequencies plays a functional role in sensory, motor and cognitive processes (Lakatos et al., 2005; Panzeri et al., 2010; Ng et al., 2013). However, the functional role of phase in cortical motor control has received much less attention (but see Miller et al., 2012) than in the domain of sensory processing.

An ongoing debate in decoding movement parameters from neuronal signals is which of these are in fact represented (encoded) in the brain. Studies of SUA revealed a “plethora of correlations” (Todorov, 2000) with various movement parameters including muscle activity (Fetz and Cheney, 1980), direction (Georgopoulos et al., 1982) and magnitude of movement velocity (Schwartz and Moran, 1999), arm position (Kettner et al., 1988), acceleration (Hore and Flament, 1988). Similarly, neuronal populations signals contain information about speed (Jerbi et al., 2007), position and velocity (Pistohl et al., 2008), as well as movement direction (Ball et al., 2009). This issue is further complicated, because—depending on the behavioral paradigm—many of the movement parameters are correlated with each other (Stark et al., 2009). Thus, position, velocity and acceleration were analyzed separately and compared in the present study.

We addressed the following questions regarding the role of phase information in decoding movement position, velocity, and acceleration from the LFC: Is the accuracy of decoding based on phase substantially higher than that based on the magnitude of the spectral components? Can the contribution of LFC phase to continuous movement decoding be understood assuming a simple model, in which the LFC contains a time domain correlate of the trajectory (or its derivatives) that “copies” the movement? To address these issues we engaged human subjects with ECoG implantations in a game-like, one-dimensional, continuous motor task and analyzed hand movement-related ECoG signals recorded from sensorimotor cortical areas in the frontal and parietal cortex with a novel decoding algorithm.

## Methods

### Subjects

Three patients participated in the study after giving their informed consent. All of them had multiple ECoG grids subdurally implanted during pre-neurosurgical evaluation of intractable pharmaco-resistant epilepsy. The study was approved by the University Clinic's Ethics Committee. Information on these three subjects and their implantations is summarized in Table 1.

**Table 1:** **Subject information**.

	**Age**	**Sex**	**Handedness**	**Grid location**	**Strip and depth electrodes location**	**Seizure onset**
S1	47	F	Right	64-contact grid right frontal	3^*^6-contact strip right prefrontal; 2^*^4-contact strip fronto-orbital; 3^*^4-contact strips interhemispheric	Right dorso-lateral prefrontal
S2	46	M	Right	64-contact grid right temporo-frontal	2^*^12-contact and 1^*^4-contact strips right prefrontal lateral; 1^*^6-contact and 1^*^4 contact strips right fronto-basal; 3^*^4-contact strips interhemispheric	Cryptogenic
S3	50	M	Right	64-contact grid right fronto-lateral	2^*^4-contact strips right fronto-basal; 1^*^12-contact strip fronto-lateral; 4^*^4-contact strips interhemispheric	Right fronto-lateral

### Task

**Figure 2:**
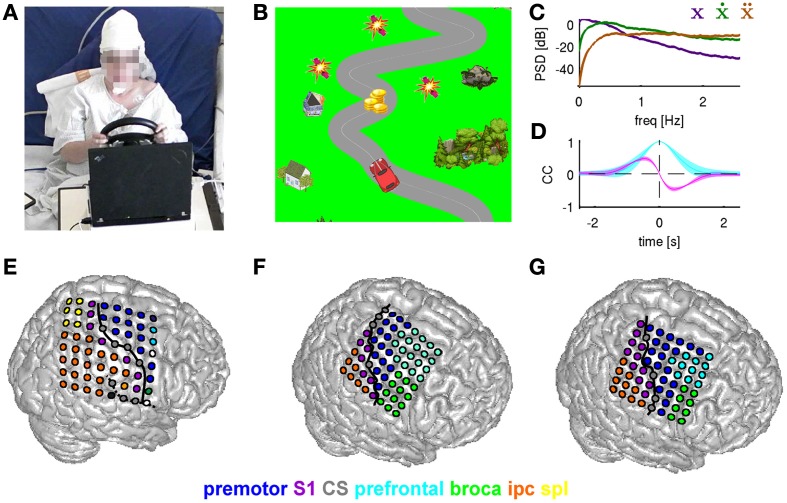
**Motor task and ECoG grid implantations. (A)** Subject S1 driving the car with a steering wheel. The paradigm (car racing game) was presented to the subjects on a laptop computer placed in front of them. **(B)** Paradigm example. Snapshot of the car on a road with objects to be collected (coins) and avoided (dynamites). Non-interactive background objects included houses and trees. The subject controlled the car in the horizontal (left/right) dimension. **(C)** Power spectrum density (PSD) for movement position (purple), velocity (green), and acceleration (brown). **(D)** Autocorrelation function of the movement position (cyan) and cross-correlation between position and velocity (magenta). **(E–G)** Anatomical assignments of ECoG electrodes to anatomical areas (for details see Methods) of subjects S1–S3, respectively. Anatomical areas are color-coded. The electrode grid is rendered on the surface of a standard brain (from SPM5). Solid black line: central sulcus determined from individual post-implantation MRI scans.

In the present study, we investigated decoding of continuous movement trajectories in one dimension, namely a horizontal movement velocity (i.e., left/right). For this purpose we designed a game-like motor paradigm, which gives the subjects a more relaxed and enjoyable experience than the stereotypical trial-by-trial movement repetitions and more nearly approximates a real life application. As illustrated in Figure 2A, subjects controlled a car on a road using a commercially-available steering wheel designed for car racing computer games (Ferrari GT Experience, Thrustmaster, La Gacilly, France). Subjects were presented with a 2D top view (Figure 2B), where the car was held in a constant vertical position and the background of the game, including the road, was sliding downwards at a constant speed of 150 pixels/s, creating an illusion of driving forwards. Importantly, the car was only controlled in the horizontal dimension via the steering wheel. The deflection of the steering wheel to either side was linearly translated into the car's horizontal position. The subjects were asked to use both hands for steering. Game-like features that were added to the paradigm were reward objects to collect (coins, four-leaf clovers), obstacles to avoid (dynamite, bombs, black cats), road splits and background objects (houses, trees). The game also had the sound of a running car engine and when rewards/obstacles were hit, additional sound effects were produced.

The characteristics of subjects' motor behavior during the game as reflected in the mean power spectrum density (PSD), computed across all subjects and all sessions of the car's (and thus, also of the movement) horizontal position, velocity and acceleration, are shown in Figure 2C. The course of the road was obtained by computing low-pass filtered white noise (3rd order Butterworth with 1.5 Hz cutoff frequency and zero phase-shift), except for the road splits (see above). The autocorrelation of the car's position (Figure 2D, cyan curve) shows that the continuous movement was correlated on a small time scale (e.g., for velocity *CC* = 0.5 at 0.2 s time lag), but not on larger time scales (>1.5 s) and also necessitates a cross-correlation peak between the trajectory derivatives (e.g., position and velocity in Figure 2D).

**Figure 3:**
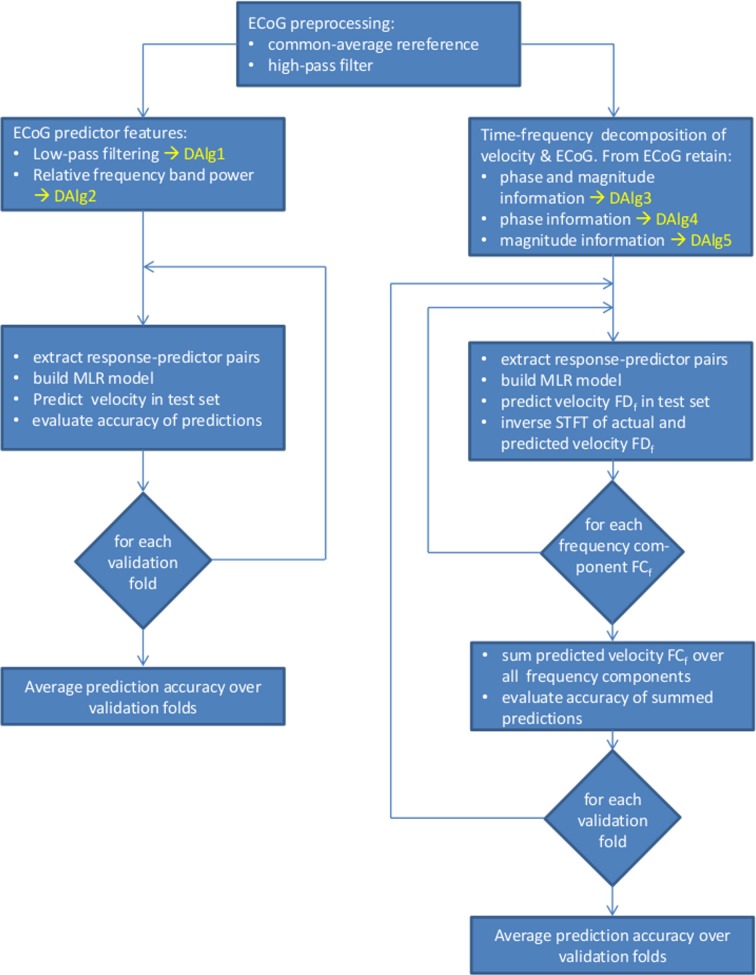
**Overview of the data processing and decoding algorithms (DAlg) used in the present study. Left side**, decoding using as predictors the time domain LFC of the ECoG (DAlg1) or the time-resolved relative power within a selected frequency band (DAlg2). **Right side**, decoding in a complex-valued, time-frequency domain with the ECoG predictors having the phase and/or magnitude information (DAlg3–5).

The aim of the game was to achieve the highest score possible. The current score was presented to the subject in the upper right corner of the computer screen. The subject was instructed (1) to stay on the road (the middle of the road was marked with a thin white line), as deviations from the road by more than 10% of the screen width resulted in a loss of points, (2) to collect reward objects on the road (resulting in plus points, for an example see the coins on the road in Figure 2B), and (3) to avoid colliding with obstacles (minus points, see dynamites in Figure 2B), even at the cost of temporarily leaving the road. One session of the game lasted 5 min, the beginning and the end were clearly indicated by start and finish flag, respectively. We analyzed 4 sessions for each subject.

### ECoG and trajectory recordings

The ECoG grid electrodes had a 4 mm diameter and 10 mm center-to-center inter-electrode distance. The site of implantation, based only upon clinical evaluation needs, included parts of hand/arm motor cortex in all three subjects (Figures 2E–G). The ECoG data were digitized at a sampling rate of 2.5 kHz (AC441-01 Neuvo amplifier, Compumedics Limited, Abotsford, Australia). Acquisition of the movement data and display of the game paradigm was performed with the Freiburg BMI Software (Milekovic et al., 2012). Subsequent data analysis was performed using MATLAB (version R2011b, The MathWorks Inc., Natick, MA). In S1, two broken channels (marked in white in Figure 2E) were excluded from the analysis.

The ECoG was synchronized with the recorded data from the game steering wheel and downsampled to 0.5 kHz. Raw ECoG recordings were re-referenced to a common average and detrended using a high-pass filter (Butterworth, 4th order, zero phase-shift) with lower cutoff frequency of 0.1 Hz. Subsequently, the data were normalized to unit standard deviation for each channel and session.

The raw, one-dimensional horizontal position tracker data, sampled at 1.0 kHz, were also downsampled to match the 0.5 kHz sampling rate of the ECoG and smoothed with a Savitzky-Golay filter (window size 0.250 s, 2nd order, corresponding to a low-pass cutoff of approximately 5 Hz) for better estimation of the derivative. The one-dimensional velocity (or acceleration) was estimated using a five-point derivative approximation of the smoothed horizontal car position (or velocity, respectively), (Abramowitz and Stegun, 1970). Subsequently, the movement data were also normalized to unit standard deviation for each session. Importantly, the movement position, velocity and acceleration used in the present study was a 1D vector variable, where the direction was indicated by its sign (negative—leftward, positive—rightward). Note that the parameter examined in the present study, in the case of velocity, was *not* the speed (= absolute value of the signed velocity).

### Electrical stimulation mapping and channel assignment to anatomical areas

During the clinical evaluation, each individual implanted electrode was electrically stimulated to produce a functional mapping of the cortex underneath the electrode. This was done by using an INOMED NS 60 stimulator (INOMED, Emmendingen, Germany) with 7 s trains of 50 Hz square pulses of alternating polarity, with gradually increasing amplitude either up to induction of sensory/motor responses of the subject or up to 15 mA pulse amplitude.

Moreover, individual electrode contacts were assigned to the cortical anatomical areas (AAs) (Pistohl et al., 2012) based on subject-specific post-implantation MRI scans. In short, in each subject, full head coverage structural MRI (T1 MPRAGE sequence) with a 1 mm^3^ resolution was acquired after the grid was implanted. Motor cortices were identified according to anatomical landmarks (Steinmetz et al., 1989; Rumeau et al., 1994; Yousry et al., 1997), individual locations of central and lateral sulci were used to assign electrodes to the frontal, parietal and temporal lobes. Further, a probabilistic atlas system (Eickhoff et al., 2006; SPM Anatomy Toolbox, version 1.7b) was used to assign each electrode to an anatomical area based on the atlas' maximum probability underneath the electrode contact. For visualization, the ECoG grids were rendered on the surface of a standard brain (SPM5) because segmentation of the complete cortical surface was not possible based on post-implantation MRIs due to electrode void artifacts. For the assignment to AAs and the location of the sulci, see Figures 2E–G. Note that with this anatomical assignment procedure, contacts in the region of the hand/arm area along the central sulcus (CS) are assigned to premotor cortex, because primary motor cortex is defined as being identical to BA4 which is entirely buried within the CS in this region of the brain. Electrical stimulation results were not used in this anatomical assignment procedure, which is more objective than previous assignments where the probabilistic anatomical information was not yet incorporated in the ECoG anatomical assignment procedure (e.g., Ball et al., 2009). The results of electrical stimulation mapping very well confirmed the anatomical definition of premotor cortex.

### Decoding model

Linear decoding methods were employed in many previous studies for decoding continuous movement, such as population vectors (Georgopoulos et al., 1982), Kalman filters (Wu et al., 2006; Pistohl et al., 2008), multiple linear regression (MLR) (Paninski et al., 2004; Georgopoulos et al., 2005; Bradberry et al., 2010). In the present study, we used MLR for decoding of 1D continuous movement position, velocity and acceleration (where the sign of the, for example, velocity indicated the direction of the movement—left/right).

The *response* (dependent, predicted variable) was the movement parameter, whereas the ECoG signal features were the *predictors* (independent variable). The formulation of the regression problem consists of forming the *response—predictor pairs* [Equation 1]. In all following analyses, we formed the predictors *x*_*k*_(*t*_*i*_ − τ) by taking simultaneous samples of ECoG features from selected channels (i.e., one sample per selected channel *k*) at a time lag τ to build one MLR decoding model β(τ).

(1) $$\left(\begin{array}{c} y(t_{1})\\ \vdots \\ y(t_{n}) \end{array}\right)=\left(\begin{array}{cccc} 1 & x_{1}(t_{1}+\tau ) & \ldots & x_{P}(t_{1}+\tau )\\ \vdots & \vdots & \ddots & \vdots \\ 1 & x_{1}(t_{n}+\tau ) & \cdots & x_{P}(t_{n}+\tau ) \end{array}\right)\left(\begin{array}{c} \beta_{0}(\tau )\\ \vdots \\ \beta_{P}(\tau ) \end{array}\right)+\left(\begin{array}{c} \varepsilon (t_{1})\\ \vdots \\ \varepsilon (t_{n}) \end{array}\right)$$

where *t*_*i*_, *i* = 1,…, *n* is the observation at the *i*-th time step, *y*(*t*_*i*_) is the response, *x*_*k*_(*t*_*i*_ − τ) is the *k*-th predictor at time step *t*_*i*_ with a time offset τ, where *k* = 0, …, *P* (*P* being the number of selected ECoG channels). β_*k*_(τ) is the *k*-th regression coefficient and ε(*t*_*i*_) is the residual error.

The *time offset* τ in [Equation 1] is an important parameter which we systematically varied in our analysis over the interval [−3.5, 3.5] s. As in other studies (e.g., Acharya et al., 2010), τ < 0 in our study reflects that neuronal activity precedes the movement, thus, indicating that ECoG features are truly “*predictive*.” In case of τ > 0, the ECoG samples occur after the velocity samples and are therefore, “*postdictive*” in the decoding sense.

This approach with only one feature sample per channel at the same time offset for all selected channels enabled us to track the unfolding of the decoded information in the time-offset domain (similar to Pistohl et al., 2008) and allowed us to uncover crucial differences in decoding magnitude and phase information (see Results).

### Prediction evaluation

The whole data sets (4 sessions with 5 min duration per subject) were split into 30 continuous data sections (folds), each of 40-s duration, which were used as validation folds for the predicted movement parameter. Response-predictors feature pairs were extracted in discrete time steps of 100 ms. The decoding performance was assessed by 30-fold cross-validation, where 29 folds (more than 19 min of recording time) were used as training sets for model building [estimating regression coefficients β(τ)] and the remaining movement validation fold as a test set for the model's velocity prediction (see [Equation 2]). This was repeated 30 times, such that each continuous velocity data section was used as test set exactly once. The quality of the prediction was evaluated with the use of the correlation coefficients (CC) between the estimated (predicted) and the actual velocity traces obtained from all test folds.

(2) $$\hat{y}(t)=\sum_{k=0}^{P}x_{k}(t+\tau )\beta_{k}(\tau )$$

### ECoG decoding features

Here we propose to use ECoG signal representations in a complex-valued time-frequency domain [for example by time-resolved Fourier transformation (FT)] to unfold the phase and magnitude values of the signal (see Figure 1). Importantly, we make a clear distinction between the *amplitude of the LFC* (the value of the low-pass-filtered ECoG signal oscillations in the time domain) and the *magnitude of the Fourier descriptors* at a given time and frequency (the absolute value of the complex numbers representing the FT in the time-frequency domain). An overview of the decoding algorithms is given in Figure 3.

The LFC as used in previous studies is a time-domain signal obtained by low-pass filtering. In spite of the widespread use of LFC in BMI research, there is considerable variability in the frequency bands and filters used across studies. With respect to the LFP, for instance, Mehring et al. (2003) used a smoothed signal (Gaussian kernel, 125-ms width, corresponding to a low-pass cutoff of approximately 15 Hz). In ECoG, the data was smoothed with Savitzky-Golay filters (2nd-order, 0.5-s width, corresponding to a low-pass cutoff of approximately 2.5 Hz) (Mehring et al., 2004; Pistohl et al., 2008; Ball et al., 2009). Several other ECoG studies (Schalk et al., 2007; Kubánek et al., 2009; Acharya et al., 2010) applied a running average, which, for example, Schalk et al. (2007) used with a window size of 333 ms (cutoff of approximately 2.5 Hz) and referred to the LFC signal as the “local motor potential.” In the following, we will, however, use the term LFC. A similar variability with respect to the exact LFC frequency bands and filters used also exists in studies utilizing non-invasive brain signals (Jerbi et al., 2007; Waldert et al., 2008; Bradberry et al., 2010).

The instantaneous phase at each time point of a narrow-band signal, that is, a signal dominated by a single oscillatory component, can be derived by the Hilbert transform and the analytical signal representation (Aertsen and Johannesma, 1980; Johannesma et al., 1981; Boashash, 1992). The LFC used in previous movement decoding studies, however, is not typically a narrow-band signal, as the maximum frequency of its magnitude envelope spectrum may be larger than the lowest frequency in the spectrum of the LFC signal itself. Such broad-band signals may contain multiple oscillatory components at different frequencies, each with a different phase. In this situation, time-frequency representations of the broad-band LFC, such as obtained by Fourier or Wavelet transforms, are useful to derive the signal phases of the different frequency components (FCs) (Figure 1). In the present study, we thus, developed an approach utilizing time- and frequency-resolved phase information for decoding continuous movement from broad-band LFC.

#### LFC in the time domain

This analysis utilizes the time domain LFC of ECoG signals (DAlg1, Figure 3). The prediction feature vector consisted of simultaneous samples from selected channels and, thus, used the amplitudes of the ECoG LFC potentials at one specific point in time (see [Equation 1]). The response for decoding was the low-pass filtered position, velocity or acceleration (5th order Butterworth with 4.0 Hz cutoff frequency and zero phase-shift), the LFC of ECoG channels served as predictors, also extracted by applying a low-pass filter (3rd order Butterworth with 1.5 Hz cutoff frequency and zero phase-shift). The upper bound cutoff frequency of the low-pass LFC filter was determined by a systematic search up to 5 Hz (see also section Choice of Frequency Bands).

#### Relative power in different frequency bands

The short time Fourier transform (STFT), with a time step of 100 ms and 2-s Hanning window, was used to compute the power of the ECoG signal in different frequency bands (DAlg2, Figure 3). The relative power of the spectra were computed with respect to a baseline value (Rickert et al., 2005), defined here as the mean frequency-bin power across each session. The relative power of a given frequency band (*f*_1_ − *f*_2_) was computed as an average over the band. This procedure avoids underestimation of the power from higher frequency bins within a defined frequency band due to the 1/*f* power decay (Miller et al., 2009). The resulting time series of the relative band power were used to predict the low-pass filtered position, velocity or acceleration (same as in section LFC in the Time Domain).

#### Fourier descriptors of short-time fourier transform

Here, we describe an algorithm with novel features using the *explicit phase information*. For simplicity, we refer here only velocity decoding, but the same approach also applies for the position and acceleration time-series. The method described below (see also DAlg3–5, Figure 3) is related to image reconstruction techniques using FT, where by taking only a first few *Fourier descriptors*, it is possible to reconstruct the image with a certain loss of detail (Persoon and Fu, 1977). We apply this basic idea to a time-series signal, for which, due to its non-stationarity, the FT was computed in time-resolved fashion. The resulting Fourier descriptor time-series of (for example!) velocity were predicted from those of the ECoG signal. Hence, both the decoding model and the prediction step were formulated in Fourier space and the predicted Fourier image of the velocity trace was subsequently mapped back to the time domain by inverse FT.

In more detail, the responses were the *time series of Fourier descriptors* of the velocity and the predictors were the *time series of Fourier descriptors* at the same frequency of selected ECoG channels. Fourier descriptors are the complex-valued coefficients obtained from FT and are associated with the carrier frequency *f* of the trigonometric function, see [Equation 3], there corresponding to the term *S*(*T*, *f*). The *time series of the Fourier descriptors at frequency f* was obtained by time-frequency decomposition using the STFT.

(3) $$S(T,f)=\int_{-\infty }^{+\infty }s(t)w(t-T)e^{-2\pi jft}dt$$

**Figure 4:**
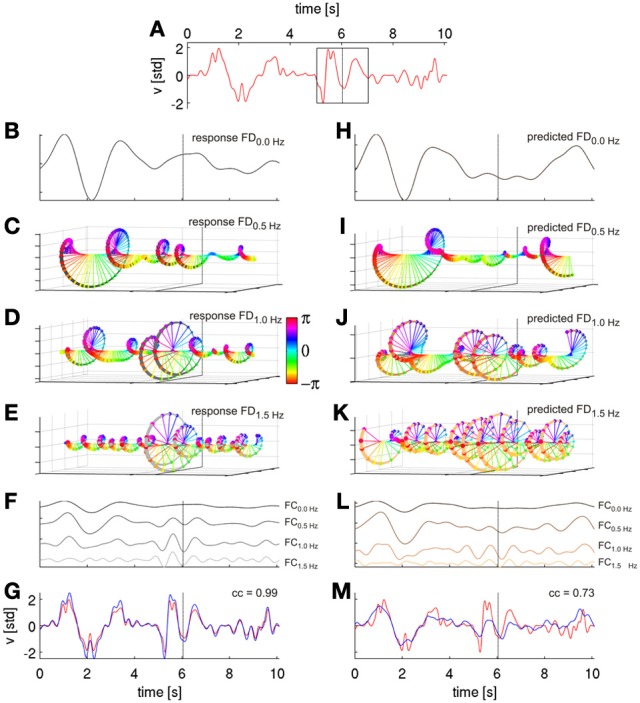
**Signal reconstruction from Fourier descriptors (FDs).** FDs were derived by short time Fourier analysis (STFT) and are illustrated based on movement velocity data from a cross-validation fold of subject S1. **(A)** Ten seconds of decoded velocity (with direction indicated by its sign), a 2-s Hanning window around time *t* of the STFT (black rectangle). The dotted vertical lines in all following plots indicate values obtained (or predicted) from this time step. **(B)** FD time series of the first frequency bin *f* = 0.0 Hz (FD_0.0 Hz_), i.e., the real-valued DC component. **(C)** FD time series of the second frequency bin, *f* = 0.5 Hz, i.e., the complex-valued FD_0.5 Hz_. FD(*T*, *f*) at different time points *T* are color-coded based on the phases of the complex values. **(D)** Third frequency bin, *f* = 1.0 Hz, FD_1.0 Hz_ **(E)** Fourth frequency bin FD_1.5 Hz_. **(F)** Reconstruction of velocity frequency components at frequencies *f* (FC_*f*_) of the first four FD_*f*_ obtained from their inverse STFT (iSTFT). The FCs are plotted with different offsets for better visualization. **(G)** Reconstruction (blue) of original (red) velocity as shown in **(A)** as a sum of the first ten FC_*f*_, with a correlation coefficient of 0.99. Thus, the sum of the FCs converges back to the original signal; the algorithm, hence, worked as intended. **(H–K)** First four Fourier descriptors of the horizntal velocity as predicted from ECoG FD_*f*_ features with time offset τ = 0 s (using DAlg3, see Figure 3). **(L)** Reconstruction (by iSTFT) of predicted velocity FC_*f*_ from the first four FD_*f*_. **(M)** Reconstruction of predicted velocity (blue) as a sum of first ten predicted FC_*f*_ and the original velocity (red) from **(A)**. The correlation coefficient between executed and ECoG-decoded velocity was 0.73 for this particular reconstruction.

The STFT transforms a real-valued time domain signal *s*(*t*) (Figure 4A) into a complex-valued time-frequency domain representation *S*(*T*, *f*) (see Figures 1C–E and 4C–E). In [Equation 3], *j* is the imaginary unit, *T* in [s] spans the same duration of time as the original time *t* and *f* is the frequency in [Hz]. The function *w*(*t* − *T*) is a (Hanning) window function [black box in Figure 4A, for which *w*(0) = 1] centered around time *T* (vertical dashed lines in Figure 4). The width of the Hanning window, inversely proportional to the frequency resolution, was set to 2.0 s (hence, frequency resolution of 0.5 Hz) and was slid in time steps of 0.1 s.

The complex-valued time series *S*(*T*, *f*) for a given frequency *f* is referred to as a *time series of Fourier descriptors at frequency f* (and further abbreviated as FD_*f*_) of the signal *s*(*t*), where the first frequency bin *S*(*T*, 0), *f* = 0 Hz, is the real-valued DC component (Figure 4B), whereas all higher frequency bins *S*(*T*, *f*), *f* > 0 Hz are complex-valued (Figures 4C–E). The squared magnitudes of these complex values define the spectral power and the angles define the phases of the respective FCs, respectively.

The STFT ([Equation 3]) was applied to the time series of both velocity *y*(*t*), resulting in the time-frequency series *Y*(*T*, *f*), and each selected *k*-th ECoG channel *x*_*k*_(*t*), resulting in the time-frequency series *X*_*k*_(*T*, *f*), with the magnitudes and phases of the ECoG relative to movement execution. The response-predictor pairs (see [Equation 1]) were then the complex-valued Fourier descriptors of velocity, *Y*(*T*, *f*_*s*_), and ECoG, *X*(*T*, *f*_*s*_), at the same frequency *f*_*s*_, where the various *f*_*s*_ were chosen to cover the interval of 0–4 Hz. The resulting model's regression coefficients β were also complex-valued and the prediction step (see [Equation 2]) accordingly yielded complex-valued estimates for each FD_*fs*_ (*Y*(*T*, *f*_*s*_), see Figures 4H–K). The predicted FDs were then transformed back to the time domain by *inverse STFT* (iSTFT, see [Equation 4]):

(4) $$s(t)w(t-T)=\frac{1}{2\pi }\int_{-\infty }^{+\infty }S(T,f)e^{2\pi jft}df$$

This inverse transformation returns only the windowed time series centered around time *T*. The FDs of frequencies not selected were set to zero. To reconstruct the original velocity time series, the iSTFT was repeated for all time steps and only the center time point *t* = *T* of each iSTFT step was kept, which reconstructs the original signal as *s*(*t*) = *s*(*t*)*w*(*t* − *T*) = *s*(*T*)*w*(0) = *s*(*T*). Note that to ensure that the result of iSTFT is real-valued, the complex conjugated “negative frequency” FDs need to be taken into account in the inverse Fourier transform (Johannesma et al., 1981). Note that [Equations 3 and 4] are given here for a continuous signal representation, in view of the simpler and clearer expressions. In the actual algorithms, we applied the discrete versions of these equations (cf., Lyons, 2003) to the signal samples at the discrete time-frequency grid.

In this way, it is possible to reconstruct the actual velocity trace (Figure 4F, demonstrating that our approach works as intended) and the ECoG-predicted (Figure 4L) velocity trace as a sum of its FCs, each frequency component being defined as the time series computed by iSTFT with a single frequency bin. Importantly, one can estimate the predictions of each selected FC separately. The sum of the FCs then converges to the original signal ([Equation 4], Figure 4G). Taking all FCs into account, the original signal would be completely reconstructed. If the predicted time series of velocity FDs are well estimated from the ECoG data, the sum of the predicted FCs also converges to the actual velocity (Figure 4M). Therefore, the presented algorithm offers great flexibility in the selection of FCs of interest. The algorithm can be consecutively run on all single frequency bins to determine the most informative FCs in a range of interest, but it can also use multiple FCs of contiguous or separated frequency bins simultaneously. If applied in this fashion, the proposed method allows to determine the decoding accuracies for any arbitrarily defined frequency band or selection of sub-bands. Moreover, to examine to which extent the ECoG was truly predictive (as expected for efferent motor control signals) or post-dictive (as expected for sensory feedback-driven responses) with respect to the movement velocity data, the time offset τ between the predictors and the response was explored in the analysis as well.

The complex-valued FD_*f*_ explicitly incorporates both magnitude and phase information (DAlg3, Figure 3). To disentangle the individual influence of magnitude and phase information on the prediction, we also compared the decoding using each of them separately, disregarding the other (but without changing the decoded velocity FD_*f*_).

To rule out the magnitude of the ECoG predictors, each Fourier descriptor *S*(*T*, *f*) as defined by the STFT (see [Equation 3]) was normalized by its magnitude (i.e., *S*(*T*, *f*) = *S*(*T*, *f*)/|*S*(*T*, *f*)|) at each time step *T*. Therefore, the magnitude of the FD_*f*_ was equal to 1 for all times *T* and frequencies *f*. However, the phase of the FDs was not changed. This signal feature (which we will further refer to as *phase information only*) was used by DAlg4 (Figure 3).

By contrast, to rule out phase information of the ECoG predictors, all phase angles were set to zero for each *S*(*T*, *f*), but the magnitudes were preserved (that is, *S*(*T*, *f*) = |*S*(*T*, *f*)|). This is equivalent to taking the magnitude envelope of the FD_*f*_. This signal feature (which we will further refer to as *magnitude information only*) was used by DAlg5 (Figure 3).

An important property of the FT in the context of the present study is that the FT of the derivative of a function *s*(*t*) is a frequency-scaled FT, *S*(*f*), of the original function, *s*(*t*). In case of the STFT:

(5) $$FT\left[\frac{d^{n}}{dt^{n}}s(t)w(t-T)\right]={\left(2\pi jf\right)}^{n}S(T,f)$$

Thus, decoding the FCs of different trajectory derivatives yields the same results in terms of the CC (as those are scale-invariant). This holds true, however, only for the FCs themselves, but not for the overall reconstructions of the trajectory derivative (e.g., position, velocity, acceleration), which are the sum of the individual FCs (as explained above), because the FCs have different power in each case (cf., Figure 2C).

### Choice of frequency bands

The estimated trajectory derivatives were low-pass filtered at 4 Hz (5th order Butterworth with zero-phase shift) for the time domain ECoG LFC decoding (DAlg1, Figure 3), mainly to enable a comparison with the time-frequency FD_*f*_ phase and magnitude decoding (DAlg3, Figure 3, where for practical purposes only the first 10 FCs—i.e., up to 4 Hz—were reconstructed). This choice retained the raw (recorded) position (*CC* = 1.00 ± 0.00), velocity (*CC* = 0.98 ± 0.00) and most of the acceleration (*CC* = 0.70 ± 0.02) profile [mean ± standard deviation (std) CC between raw and [0–4] Hz low-pass filtered kinematic signal, over all 4 sessions of all 3 subjects].

**Figure 5:**
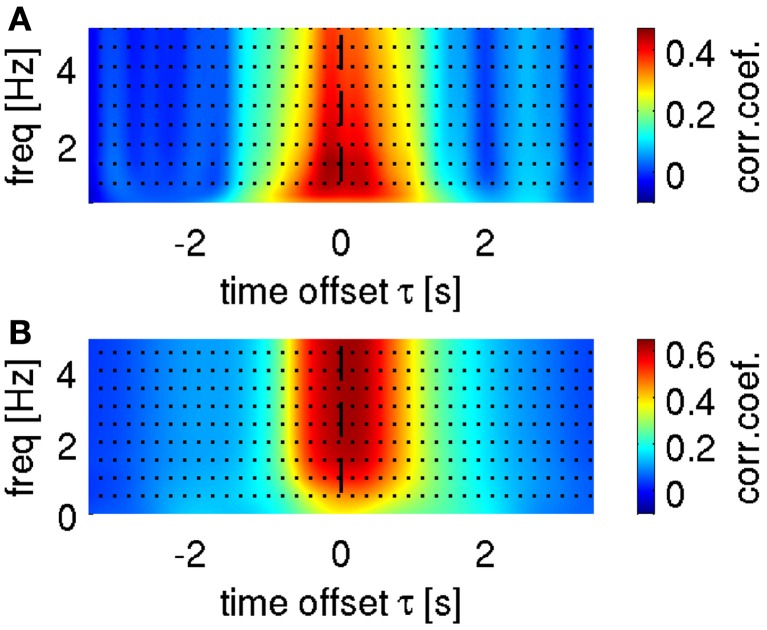
**Selection of optimal frequency ranges for velocity decoding.** Representative results are shown from subject S1. **(A)** Search for low-pass filter cut-off frequency in time-domain ECoG LFC decoding. Mean correlation coefficient as a function of upper bound low-pass filter and a time offset τ between predictors (LFC ECoG feature vector) and decoded movement velocity. The highest correlation coefficient based on the LFC was found in the frequency range [0.1–1.5] Hz in all subjects. **(B)** Cumulative sum (bottom-up direction) of predicted velocity frequency components at different carrier frequencies *f* (FC_*f*_), decoded using time-frequency domain ECoG FD_*f*_ features (DAlg3, Figure 3). Mean correlation coefficient between the cumulated predictions (i.e., FC_*f*0_, FC_*f*0_ + FC_*f*1_, FC_*f*0_ + FC_*f*1_ + FC_*f*2_, …) and actual velocity with different time offsets τ between predictors and movement velocity (as in **A**). Decoding accuracy saturated in all subjects after including the first 5 FC_*f*_.

To set the optimal cut-off of the low-pass filter for the ECoG data in DAlg1, we conducted a search over this parameter (example for velocity in Figure 5A). Consistently across all subjects, we found a global maximum in the decoding accuracy (DA) at 1.5 Hz, which was then used to illustrate decoding and tuning analysis results.

A further question is how many and which predicted FCs should be taken into account for reconstruction with time-frequency features (DAlg3, Figure 3). To address this question, we analyzed the CC between the velocity and a gradual sum of the individually predicted velocity FCs, starting with the 0-Hz DC component (Figure 5B). We found that the DA saturated already after the first 5 FCs, mainly because the higher velocity FCs (>2 Hz) had only small amplitudes and, hence, little impact on the overall velocity reconstruction (cf.,, Figures 2C, 4F). But as the DA also did not deteriorate with incorporating these higher FCs, we used all first 10 FCs (up to 4 Hz) for the reconstruction nevertheless.

### ECoG tuning analysis

The tuning analysis gives an insight into how the ECoG signal features are modulated given the measured velocity (in this section we concentrated to illustrate the role of phase only on velocity tuning). Smoothed and normalized velocity was binned to 20 bins, the sizes of which were adjusted such that each bin contained an equal number of samples. For all samples of the velocity assigned to a certain bin, a “grand mean” of the corresponding ECoG features was computed, as the mean over all *events*' means. An *event* in this analysis was defined as the continuous data segment starting at the time when the velocity time series entered the bin boundary until it left it again (and entered the next bin). For each such *event*, we extracted (with a certain time lag—see section Decoding Model) the corresponding ECoG samples and computed their mean. The final value of the tuning (the “grand mean”) in each bin is then the mean over all such *events*. This procedure avoids potential over-representation of *events* which would be disproportionally longer than others and ensures that the tuning results are representative for the entire time interval analyzed. In parallel to the decoding analysis, we also performed a time offset-resolved tuning analysis, both for the real-valued LFC and for the complex-valued FD_*f*_ features of the ECoG signal. Note that we defined time offset τ such that its negative value reflects that neuronal activity precedes the movement execution (see Methods, section Decoding Model, for further details).

## Results

### Phase-only and magnitude-only based decoding of movement parameters

**Figure 6:**
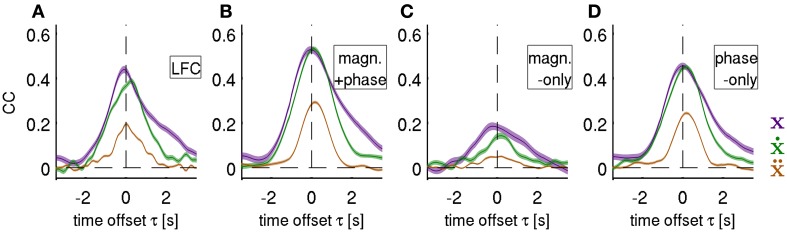
**Decoding of position, velocity and acceleration.** Results obtained from decoding all ECoG grid channels as an average over all movement test sets (i.e., 3 × 30 validation folds) for subjects S1–S3. Mean *CC* ± s.e.m. as a function of time offset τ between ECoG predictors and decoded movement position (purple), velocity (green), and acceleration (brown). Negative values of τ indicate that the ECoG preceeds movement execution. The four different features (rows of the figures) analyzed are: **(A)** *time domain LFC* (DAlg1), **(B)** *time-frequency magnitude and phase* (DAlg3), **(C)** *time-frequency magnitude-only* (DAlg5) and **(D)** *time-frequency phase-only*. (DAlg4) Decoding based on features with information from low-frequency ECoG phase was substantially better than that of the magnitude only.

**Figure 7:**
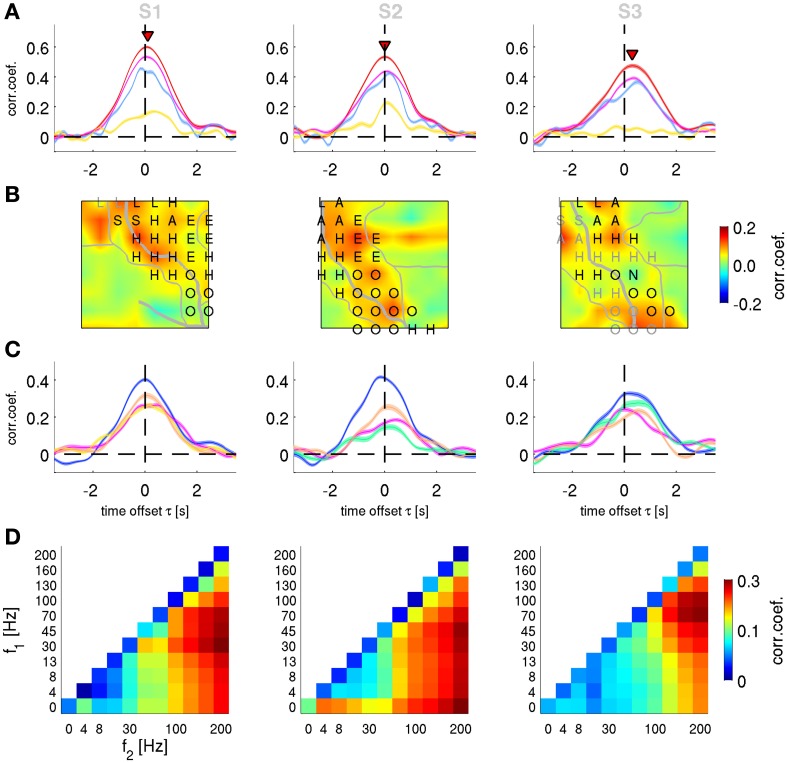
**Decoding of velocity from different signal components and anatomical areas.** Results for subjects S1–S3. **(A)** All grid channels decoding. Mean *CC* ± s.e.m. as a function of time offset τ between ECoG predictors and decoded movement velocity (negative values of τ indicating that the ECoG preceeds in time the movement execution). The 4 different features analyzed are: *time-frequency magnitude and phase* (red), *time-frequency phase-only* (magenta), *time-frequency magnitude-only* (yellow), and *time domain LFC* (cyan). Decoding accuracy of time-frequency magnitude and phase features at their peak values (labeled with red triangles) are significantly better than those of the time domain LFC. **(B)** *Time-frequency magnitude and phase* decoding using single channels and time offset τ indicated by red triangles in **(A)**. The square plots represent the ECoG grid of each subject, with marked central (and in S1 lateral) sulcus (thick white curve on the grid), division of anatomical areas (thin white lines, cf., Figures 2E–G) and the labeled electrical stimulation results (label color: magenta—motor response, gray—sensory response. H—hand, A—arm, O—oral, E—eyes, L—leg, S—shoulder, N—neck). **(C)** Decoding of channel groups based on assignment to anatomical areas (section Electrical Stimulation Mapping and Channel Assignment to Anatomical Areas in main text) using the time-frequency magnitude and phase features. Colors of the anatomical areas are the same as those in Figures 2E–G. Premotor area (in blue) provides the most accurate predictions in all subjects. **(D)** Decoding from relative power modulations in a wide range of different frequency bands. The correlation coefficient is color-coded for each frequency band (*f*_1_ − *f*_2_), as defined by the *x*- and *y*-axes. Results are shown for the time offset τ with the maximal correlation in each subjects (0.0 s, −0.1 s, and 0.2 s for subjects S1–S3, respectively). The phase of the slow oscillations was clearly more informative than any of the spectral band power features.

Using the decoding approach based on a time-frequency representation of the ECoG as described in section Fourier Descriptors of Short-Time Fourier transform, we first addressed the question whether LFC phase is indeed the major source of movement-related decodable information. By comparing results from DAlg5 (magnitude information only) and DAlg4 (phase information only) obtained from the same data set, it was possible to quantitatively assess the relative contributions of magnitude and phase separately. We compared the decoding performance taking all channels of the ECoG grid at one specific time offset τ to predict the velocity. This time offset τ was systematically varied over the interval [−3.5, 3.5] s. Note that we defined the time offset τ such that its negative value corresponds to the situation where ECoG activity precedes the movement (see Methods, section Decoding model, for further details). We found that in all subjects phase clearly proved to be substantially more informative than magnitude in all trajectory derivatives (Figures 6C,D, for direct comparison of velocity prediction in single subjects cf., yellow and magenta curves in Figure 7A). Peak correlation coefficients (CCs) between actual and predicted velocity for all subjects movement validation folds were in the range 0.46 ± 0.10 (mean ± std) for phase-only features, which was significantly higher (paired, two-sided sign test of 30 CCs from each cross-validation fold and time lag, *P* = 0.001 significance level, false discovery rate correction for multiple tests over time lags) than for magnitude-only features, where *CC* = 0.16 ± 0.12 (Table 2). Moreover, maximal DA achieved using phase only was very similar to that obtained using the time-domain LFC (DAlg1, Figure 6A, Table 2, also cf., magenta and cyan curves in Figure 7A). These findings clearly identify phase (and not the magnitude) as the major carrier of information for ECoG LFC decoding of movement velocity.

**Table 2:** **Peak decoding accuracies. CC values (mean ± std over the 3 × 30 movement validation folds of all 3 subjects), at the optimal time offset defined for each subject individually**.

	**LFC**	**Magn. +phase**	**Magn. only**	**Phase only**
Position	0.45 ± 0.15	0.53 ± 0.15	0.21 ± 0.16	0.46 ± 0.14
Velocity	0.41 ± 0.11	0.54 ± 0.10	0.16 ± 0.12	0.46 ± 0.10
Acceleration	0.20 ± 0.07	0.30 ± 0.07	0.06 ± 0.04	0.25 ± 0.06

### Decoding of movement using phase and magnitude simultaneously

**Figure 8:**
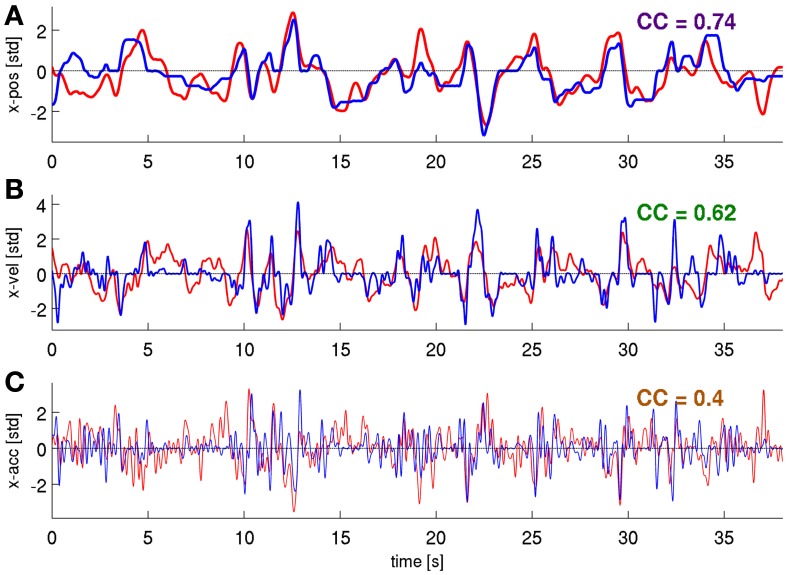
**Predictions of position, velocity, and acceleration from a representative validation fold.** Using the *time-frequency magnitude and phase* features (DAlg3, τ = 0 s). **(A)** position, **(B)** velocity, **(C)** acceleration, CCs are given for these individual examples.

We next tested whether a further increase in DA could be obtained by decoding movement velocity using phase and magnitude simultaneously (DAlg3, Figure 3). Comparison of results using all channels of the ECoG grid as predictors showed a consistent pattern for all three subjects (Figures 6B, 7A, red curves): the complex features containing information on phase and magnitude had the best performance and were significantly better than the LFC (Figure 7A, blue curves)—paired, two-sided sign test of 30 CCs from each cross-validation fold and time lag, *P* = 0.05 significance level, false discovery rate correction for multiple tests over time lags. Examples of predicted trajectory derivatives (using the DAlg3) is shown in Figures 4M, 8.

### Time-resolved decoding of position, velocity and acceleration

Decoding of position and velocity from the phase in the low frequencies yielded very similar DA for all algorithms (Figure 6, Table 2), while acceleration was consistently and significantly worse decodable. Systematically taking a feature vector as channels with only one time offset τ between response- predictor pairs and varying this offset value over an interval gives a temporal profile of the decodable information in the predictor signals with respect to the movement execution. We found, consistently, that (1) the onset occurred 2 s prior to movement, i.e., in the “prediction” stage, (2) with a peak around 0 s, corresponding to the time of movement execution, and (3) returning to zero baseline level around 2 s after movement execution, in the “postdiction” stage (Figures 6, 7A).

Notably, the temporal profile of the DA as obtained from the LFC signal appeared less smooth than that based on the magnitude and phase features. This was particularly clear in velocity decoding of subject S1, where LFC-decoding showed a double peak (Figure 7A, left), whereas magnitude and phase decoding showed a single smooth peak in the same time window. The reason for these temporal differences was further explored and clarified using single-channel tuning analyses of velocity (see below, section Single Channel Velocity Decoding and Tuning).

### Spatially resolved velocity decoding

To characterize the anatomical origin of the decodable information, we performed two sets of spatially resolved decoding analyses. We focused here only on velocity (although similar observations were made also for position and acceleration). First, we computed a single-channel based analysis for the FD_*f*_ features. The decoding algorithm (DAlg3, Figure 3) was the same as the one used in section Decoding of Movement using Phase and Magnitude simultaneously, with the exception that the predictors consisted of only one channel sample at the time offset τ of maximum performance of all channel magnitude and phase features (indicated by the red triangles in Figure 6A). Thus, for each channel, we constructed a different model and assessed its prediction individually. CC values for rejected channels in S1 (Figure 2E, white color) were interpolated from neighboring electrodes.

The result of this analysis is shown as a grid map of the CC values (Figure 7B). In all subjects, channels near the central sulcus (CS, recording from premotor and primary somatosensory areas) had an overall better performance than the channels further away from the CS.

Second, to quantify the contribution of the different AAs defined as described in the Methods (section Electrical Stimulation Mapping and Channel Assignment to Anatomical Areas), we performed a decoding analysis (DAlg3, Figure 3) using those channels assigned to each of the AAs. In all subjects, the premotor and primary somatosensory areas could be analyzed in this way, Broca's area in subjects S2 and S3, the superior parietal cortex (SPL) in S1. The remainder of the ECoG grid channels (labeled as “other”), not included in any of the aforementioned AAs, were analyzed together. Across all subjects, the premotor area showed the best decoding performance among the areas analyzed (Figure 7C).

### Relative power of frequency bands velocity decoding

The phase of high frequency oscillation (>4 Hz) cannot be readily used to continuously predict the movement velocity (the spectrum of which <4 Hz, cf.,, Figure 2C), because the phase is changing faster than the relatively slow time course of the movement velocity. Such an argument, however, does not hold when a magnitude (or power) envelope of a high frequency oscillation is considered. Thus, the relative power of the frequency bands (DAlg2, Figure 3) defined by all possible combinations of band limits from the following range were investigated: 0, 4, 8, 13, 30, 45, 70, 100, 130, 160, and 200 Hz. The optimal time offset τ was assessed individually for each subject (Figure 7D). For subject S1, the global maximum was found at τ = 0.0 s, *CC* = 0.33 ± 0.09 (mean ± std) in the 30–200 Hz frequency band; for subject S2: τ = −0.1 s, *CC* = 0.30 ± 0.10, 45–200 Hz band; for subject S3: τ = 0.2 s, *CC* = 0.27 ± 0.10, 70–200 Hz band. There was a considerable variability in the time offset values for the decoding maximum, but certain consistency for the best frequency band power, namely in broad-band high gamma (75–200 Hz)—with only the lower boundary being variable across subjects. Importantly, the LFC and/or the complex FD features yielded significantly better predictions than any of the band-limited spectral power features of the same signal.

### Frequency resolved decoding of position, velocity, and acceleration

In the results from *magnitude and phase features* (DAlg3, Figure 3) presented thus, far, the decoded variable was acquired as a sum of its predicted FCs. An additional useful property of the algorithm proposed here is that DA can be assessed in a frequency resolved way, evaluating the CC of each FC separately (CC_FC_), thus, allowing detailed insight into the most informative narrow-band FCs within the broad-band LFC. To examine the frequency-resolved profile of the CC_FC_ at higher frequency resolution, we also performed the same analysis as before, but with a broader Hanning window of a 5-s duration (corresponding to a frequency resolution of 0.2 Hz). The *time-frequency magnitude and phase* predictors were taken at time offset τ = 0 s.

From the above results (Figures 6, 7A) followed that the magnitude of the LFC contributed only marginally to the decoding of the continuous movement and the phase was clearly identified as the major source of decodable information. Similar results (i.e., phase DA >> magnitude DA) were obtained, when the only predictor channel is the kinematic signal itself (e.g., position trivially decoding position itself). Then, using the *time-frequency magnitude and phase* predictors, as expected, all FCs were perfectly predicted regardless of their spectral profile (as long as that was not identically zero).

**Figure 9:**
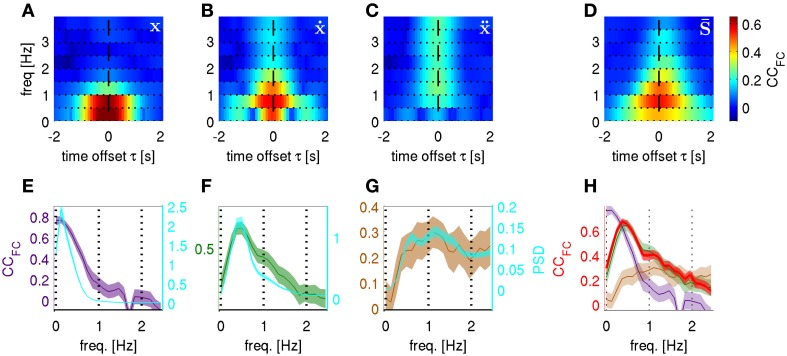
**Time-frequency resolved decoding from “kinematic copy + white noise” models and from real ECoG.** Correlation coefficients between predicted and actual frequency components (CC_FC_) computed at different carrier frequencies *f* and time offsets τ. The kinematic copy (std = 1) was summed up with white noise (std = 20). **(A)** “position + noise” model predicting position, **(B)** “velocity + noise” predicting velocity and **(C)** “acceleration + noise” model predicting acceleration. **(D)** The CC_FC_ for real ECoG data predicting trajectory derivatives, i.e., position, velocity, and acceleration (averaged over all subjects and also all trajectory derivatives, see section Frequency Resolved Decoding of Position, Velocity, and Acceleration). **(E)** “position + noise” model, CC_FC_ frequency profile (purple curve, left *y*-axis) for the *time-frequency magnitude and phase* features at time offset τ = 0 s and with higher frequency resolution (window size = 5 s) plotted against the power spectral density of the time course of position along the trajectory (PSD, cyan, right *y*-axis). **(F)** Same as **(E)** for “velocity + noise” model (green curve, left *y*-axis). **(G)** Same as **(E)** for “acceleration + noise” model (brown curve, left *y*-axis). **(H)** The CC_FC_ obtained from decoding the real ECoG data (red) plotted on top of the CC_FC_ of the 3 models in **(E–G)**. There is only a close match between the ECoG CC_FC_ and the frequency profile resulting from the “velocity + noise” model (green).

This observation led us to the hypothesis that the LFC could be understood as a close time domain copy of a kinematic variable. A somewhat more realistic scenario was constructed when white noise was added to the kinematic “copy” [thus, “copy + noise” model, kinematic signal (with std = 1) + white noise (with std = 20)]. In this case, expectedly, the FCs coinciding with the maximal power spectral density (PSD) of the movement position (Figures 9A,E), velocity (Figures 9B,F) and acceleration (Figures 9C,G), thereby having maximal signal-to-noise ratio, were also best decodable. Notably, the frequency profiles of the CC_FC_ were quite different for each kinematic “copy + noise” model (average over all trajectories of all subjects).

For the ECoG data recorded in subjects S1–S3 (last column of Figure 9) we found that the most informative FCs were consistently in the very low frequency domain between 0.5 and 1.0 Hz. As denoted at the end of section ECoG decoding features, the prediction from the ECoG of the FCs for any of the kinematic derivatives must be the same (which was indeed observed; Figures 9D,H is an average over all subjects and all kinematic derivatives). Surprisingly, the time-frequency resolved decoding of the CC_FC_ (τ, *f*) showed (Figure 9D) a very similar frequency profile as that of the “velocity + noise” model (Figure 9B), which was also confirmed in the decoding results at a higher frequency resolution (Figure 9H).

In the light of these results, it became also clear why the acceleration had a significantly lower DA than the position or velocity (Table 2). This was due to the fact that the most informative FCs were located between 0.5–1.0 Hz, while high frequencies were less informative (Figures 9D,H, *f* > 2 Hz). Hence, the high-frequency components important for acceleration reconstruction were relatively inaccurately predicted (Figure 9H, cf., red and brown curves for *f* > 2 Hz). This was not the case for position or velocity the high FCs of which had relatively little power in our motor task (Figures 9E,F, cyan curves).

### Single channel velocity decoding and tuning

**Figure 10:**
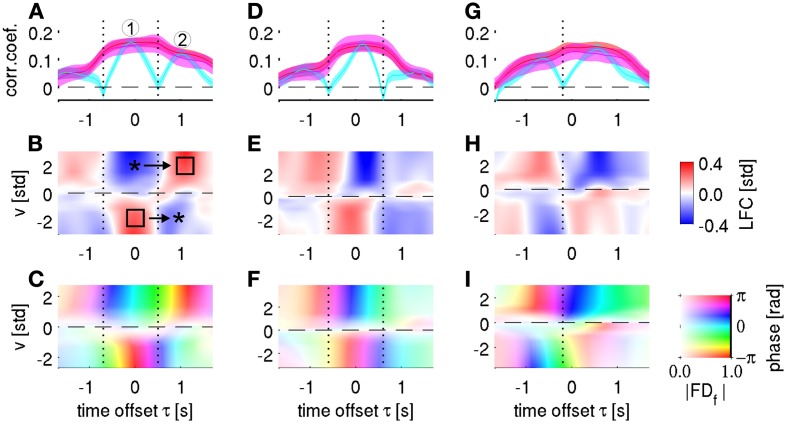
**Single channel velocity decoding and tuning analysis.** Columns represent the three selected channels from subjects S1–S3, respectively. **(A)** Single-channel decoding accuracy at different time offset τ (same notation as in Figure 5A). The three different features analyzed are: time-frequency magnitude and phase (red), time-frequency phase-only (magenta), and time-domain LFC (cyan). The selected channel corresponds to 1st row and 3rd column in the ECoG grid of subject S1 (cf., Figure 2). Note the “camel back” shape of the LFC-based decoding with two separate peaks indicated by “1” and “2.” **(B)** Velocity—ECoG LFC tuning. The *x*-axis defines the time offset τ between velocity and ECoG data, the *y*-axis defines the velocity bins *v* (where *v* > 0 for rightward movements and *v* < 0 for leftward movements). The binned ECoG LFC average is color-coded (and interpolated). Around τ = 0 s, a polarity gradient of mean LFC from positive (for *v* < 0) to negative (for *v* > 0) can be observed (left star and square, respectively). Approximately 1 s later, the polarity was opposite (right square and star). The minimum of LFC-based decoding accuracy in **(A)** as indicated by a vertical dotted line clearly corresponds to the time of polarity reversal in **(B)**, where the LFC showed little tuning. **(C)** Velocity—ECoG FD_*f*_ tuning, where *f* = 0.5 Hz. The velocity binning is identical as in **(B)**, the complex-valued FD_*f*_ response is transparency-color-coded (scaled as indicated by the color bar in the lower-right corner), where transparency indicates the magnitude of the averaged FD_*f*_ features and their phase angles, defined as arctg(Re/Im), are coded by a circular color map. At the time points of minimal LFC decoding accuracy (vertical dotted lines), the phase is still tuned, due to the polarity changes as shown in **(B)**, explaining the high phase-based DA at these time points (magenta curve in **A**). **(D–F)** same as in **(A–C)**, the selected channel corresponds to 5th row and 4th column of the ECoG grid of subject S2. **(G–I)** same as in **(A–C)**, the selected channel corresponds to 8th row and 5th column of the ECoG grid of subject S3.

To further explore the role of phase in motor decoding, we performed single channel decoding and tuning analyses. In the analyses based on all channels as described above, decoding utilizing phase showed a smoother time course than LFC-based decoding (see above, Figures 6, 7A). This difference was even more pronounced at the single channel level (Figure 10). The time offset course of LFC-based decoding typically showed clearly distinct, multiple peaks, while decoding based on phase (alone or in combination with magnitude) was much smoother (cyan vs. red/magenta curves in Figure 10A). This effect can be intuitively understood from time-resolved single channel velocity tuning of the different signal components.

Figure 10A shows an example of a velocity-tuned channel from premotor cortex of subject S1. Two peaks of DA can be clearly distinguished (marked 1 and 2, respectively) in the temporal profile of LFC-based decoding (cyan curve). Between these peaks, CCs between real and predicted velocity dropped close to zero (indicated by black dotted line). The time-resolved velocity tuning plot of the same channel (Figure 10B) reveals that during the first DA peak, rightward velocity (positive signed) was associated with a negative potential (indicated by the upper star in Figure 10B), and, likewise, leftward velocity with a positive potential (lower square). These differential LFC responses explain the good decodability at this time offset. During the second DA peak, however, the polarity of the LFC tuning was reversed (positive LFC during rightward and negative LFC during leftward movement, marked with upper square and lower star in Figure 10B). This polarity change explains the vanishing LFC tuning at the intermediate latency (black dotted line). At that same point in time, however, the signal phase was clearly tuned (Figure 10C)—due to the opposite direction of polarity change: from negative to positive for rightward and from positive to negative for leftward movements, respectively. As a consequence, velocity is still well decodable at this intermediate time point if phase information is being used (with or without additional magnitude information, red and magenta curves in Figure 10A) and the resulting temporal profile is smooth. Similar effects were observed in many motor-cortical channels of all subjects (Figure 10, remaining panels).

## Discussion

It has been previously suggested that phase information might be particularly important for motor decoding (Jerbi et al., 2007). But it was unclear if the phase is the only major source of the decodable information or, alternatively, whether magnitudes substantially contribute as well (Ball et al., 2009). In the present study, we applied a decoding algorithm enabling us to address this question directly by decoding from either phase or magnitude information alone, from their combination, from the low-pass filtered ECoG, as well as from band-limited spectral power. Our findings clearly show that the ECoG LFC phase is indeed much more informative than magnitudes (Table 2, Figures 6, 7A) and that the frequency profile of decodable information closely matches the power of the individual velocity FCs (Figure 9H). Both of these observations are consistent with the assumption of a “copy”-like representation of movement-related information, in our case velocity, in the LFC of neuronal population activity signals such as the ECoG. The decoding accuracies from spectral power in different frequency bands (Figure 7D) were significantly lower than that of the phase of slow oscillations. The best DA was achieved from broadband high gamma (70–200 Hz), *CC* = 0.30 ± 0.10 (mean ± std). Such results are consistent with previous reports (Pistohl et al., 2008; Ball et al., 2009). Combination of different features (e.g., high gamma + LFC) is a further promising strategy to increase the overall DA needed for practical BMI application.

In previous ECoG studies using similar continuous motor tasks as ours, Schalk et al. (2007) reported an average CC around 0.5 and Pistohl et al. (2008) reported values around 0.4. Our decoding results (Table 2) had the best DA for velocity (mean ± std *CC* = 0.54 ± 0.10) and are, thus, comparable with respect to the achieved accuracy, indicating that using the time-frequency domain *magnitude and phase features* (DAlg3, Figure 3) can be useful for motor decoding and might also increase the performance of online BMIs based on similar approaches (Milekovic et al., 2012).

The time domain and frequency domain representations of a signal are mathematically equivalent. Hence, with optimal decoding methods, the resulting DA should be the same in the two cases. In the present study, we compared LFC decoding based on temporal data from a single time bin with the complex-valued frequency features from the same time bin, as we aimed at delineating the separate roles of phase and magnitude. We thus, performed the decoding based on all available time points individually, which enabled us to relate the time offset profiles of phase and LFC encoding and decoding with maximal temporal accuracy (see below).

The temporal evolution of the phase-based DA, obtained by systematically varying the time offset τ between the ECoG predictors and velocity response, showed a relatively smooth time course with the global maximum at a time close to movement execution (Figures 6, 7A). This basic temporal profile was observed for decoding based on all electrodes (Figure 7A), as well as for individual AAs (Figure 7C). The shape of the DA curve reflects the underlying neuronal processes, including motor preparation and sensory processing, as well as the auto-correlation of the kinematic response signal (Figure 2D), the low frequency characteristics of the features used as predictors, and the processing of the decoded features (zero phase shift filtering, windowing, etc.). Notably, this smooth time course was not obtained with LFC-based decoding, yielding “camel-back” profiles, most prominently in the single channels (Figure 10). These multiple peaks could be taken to suggest distinct stages of neural processing, while phase decoding rather indicates a smooth, continuous evolution of movement-related information.

Pistohl et al. (2008) also showed the unfolding of the decodable information by taking one and the same time offset for all channels as in the present study. However, their analysis was restricted to the prediction phase (negative values of time offset τ in our study) only. We intended to study the whole temporal profile and, hence, also used the ECoG from the “postdiction” phase (i.e., velocity at a given time point is decoded from ECoG activity at a later time point). We found that maximal accuracy was obtained when decoding velocity from close-to-simultaneous ECoG. In S3, the velocity DA peak (i.e., the time offset with maximum CC, see red triangles in Figure 7A) was even slightly shifted into the “postdiction” phase. This temporal property is important for the construction of closed-loop BMIs because, obviously, one cannot use information from the “postdictive” part in real-time decoding. The present findings suggest that the offset between ECoG features and decoded velocity should be minimized.

The spatial distribution of the decodable information had a plausible topography. Individual channels with highest DA were well aligned with the motor areas according to electrical stimulation mapping (Figure 7B). The anatomical area with best performance was the pre-central motor area (Figure 7C), in line with findings from previous ECoG decoding studies (Ball et al., 2009).

Another question that received much attention in ECoG decoding studies is which frequency band is most informative. The exact frequencies within the low-frequency range which provide most information have, however, not yet been determined. The decoding algorithm proposed in the present study is based on decoding the Fourier descriptors in the time-frequency domain and is, thus, suitable to disentangle the DA obtained from individual FCs. We found that, consistently in all subjects, the very low FCs between 0.5–1.0 Hz were the most informative, with the DA peak around 0.5 Hz (Figures 9D,H). The possibility of such frequency-resolved examination has practical advantages for the selection of the FCs to be used for later reconstruction and/or optimal filter selection.

Polarity changes were a prominent feature in the velocity-ECoG tuning (Figure 10). These time points of polarity changes are of special interest because, here, phase but not LFC decoding was informative. This can be understood because LFC decoding required a linear tuning of the LFC signal to velocity, as clearly seen for those time lags where LFC activity was most informative (Figures 10A,D,G): here, the LFC had opposite polarity for the two different movement directions. This linear tuning vanished at the time points of polarity reversal (vertical dotted lines in Figure 10) and, hence, LFC-based decoding failed, while the phase remained tuned and decodable (Figures 10A,D,G, red and magenta curves). Taking more than one channel into the LFC prediction feature vector makes this effect less pronounced, but still visible (Figure 7A, cyan curve), as the polarity reversals occurred at different time offsets across channels (cf., Figures 10A,D,G).

In summary, the findings of the present study show that during a continuous motor task, phase and not magnitude substantially contributed to movement kinematics decoding, as previously assumed but not quantitatively tested, and that the frequency profile of DA matched well with the shape of the PSD of the movement velocity. These findings are consistent with the assumption that there is a close copy of the velocity embedded in the multi-channel time-domain ECoG data, and that there are no additional magnitude-based mechanisms encoding velocity in the LFC frequency range, as illustrated by a simple “velocity + noise” model. In following studies it would be interesting to test to which extent also movement data with other frequency compositions can be closely “copied” by the LFC, such as faster movement with their maximal frequency content in higher frequencies, or even superpositions of slow and fast movements resulting in more than one spectral peak. Would the frequency profile of the reconstructed FCs remain similar as reported in this study or would it match the PSD profile as predicted by the “velocity + noise” model? With the methods proposed here it would also be interesting whether there are other tasks or movement parameters where magnitudes contribute substantially to movement decoding, i.e., how general the predominance of movement-related information in LFC phase is in ECoG decoding.

### Conflict of interest statement

The authors declare that the research was conducted in the absence of any commercial or financial relationships that could be construed as a potential conflict of interest.
